# Exploring experiences of swimming and aquatic exercise in individuals with long term health conditions: results from Swim England’s ‘England Swims’ survey

**DOI:** 10.1093/eurpub/ckag075

**Published:** 2026-07-10

**Authors:** Lewis Wright, Fiona Moffatt, Kerry Watkiss, Andrew Power, Dominic O’Connor

**Affiliations:** School of Health Sciences, University of Nottingham, Queen’s Medical Centre, Nottingham, United Kingdom; School of Health Sciences, University of Nottingham, Queen’s Medical Centre, Nottingham, United Kingdom; Swim England, Sport Park, Loughborough University, Loughborough, United Kingdom; Swim England, Sport Park, Loughborough University, Loughborough, United Kingdom; School of Health Sciences, University of Nottingham, Queen’s Medical Centre, Nottingham, United Kingdom

## Abstract

This study analyses data from Swim England’s *England Swims* survey to explore perceptions of swimming and aquatic exercise among individuals with long-term health conditions (LTHCs) and to identify barriers to participation. This cross-sectional study involved analysis of responses collected during the 2022 ‘England Swims’ survey. Demographic information was summarized using descriptive statistics. Participant responses were summarized descriptively. Free text responses were analysed thematically according to Miles and Huberman techniques of labelling, coding, categorizing and theme development. Constant comparative techniques were used to ensure all perspectives were represented in the analysis, and deviant cases examined. Of the 6608 individuals who opened the survey, a total of 4615 responses were received (70% response rate), of these, 816 (18%) reported having a LTHC. Of those with LTHCs, 606 (74%) reported that they could swim, 212 (26%) reported they could not. The three most common barriers reported amongst non-swimmers were a lack of disposable income (*n* = 208, 43%), it is too expensive (*n* = 191, 40%), and a lack of motivation (*n* = 178, 37%). Analysis of the free text responses identified four themes related to swimming: (i) Confidence, (ii) Fear of discrimination, stigma or unwanted attention, (iii) Availability, and (iv) Unwelcoming environment. The findings reveal that despite generally positive perceptions of swimming, multiple barriers prevent many from engaging. Targeted interventions to make swimming more accessible and appealing to individuals with LTHCs are needed. Future research would be valuable for informing policy decisions and should explore the effectiveness of specific interventions aimed at addressing these barriers.

## Introduction

Globally, large numbers of adults (27.5%) [1], and adolescents (81%) [2] do not meet current physical activity (PA) recommendations. In the United Kingdom (UK), physical inactivity is associated with one in six deaths and costs £7.4 billion annually [3], while over a third of adults live with a long-term health condition (LTHC) [4], a major challenge for health and social care [5].

Regular PA can reduce risk of morbidity and premature mortality [6] and supports rehabilitation and management of LTHC’s [7–10]. In the World Health Organisations (WHO) 2020 guidelines for PA and sedentary behaviour [11], they explicitly highlighted the benefits of PA for those with LTHC’s, a group in which physical inactivity levels are likely higher [12], for improving health outcomes.

**Figure 1.**
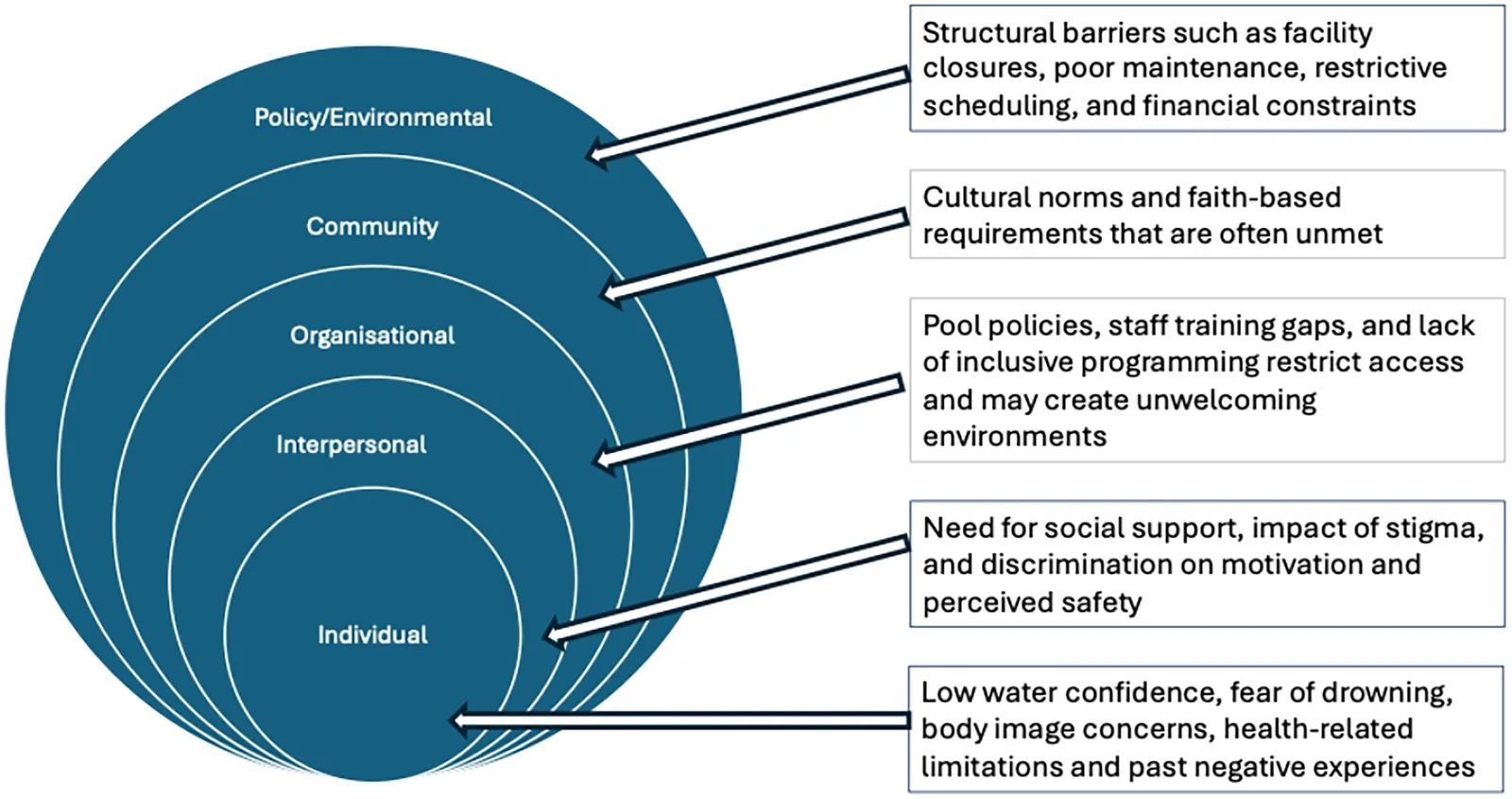
Ecological model of health behaviour.

Physical activity promotion is an important goal for public health and public policy. However, for those with LTHCs this can be challenging due to fears of symptom aggravation, injury or death. Many require reassurance that PA benefits outweigh the risks [13]. Evidence-based, safe activities are essential for managing LTHCs and improving health and fitness. Compared with many land-based activities, swimming offers low-impact, whole-body aerobic exercise with reduced joint loading and falls risk [14], offering an environment for people with LTHCs to exercise safely and effectively with benefits in quality of life, balance and strength reported [14]. As a lifelong activity, swimming supports social inclusion, reduces anxiety and depression [15] and improves aerobic fitness, making it one of the most popular forms of PA worldwide [16]. However, aquatic exercise provides reduced weight-bearing stimulus compared with land-based walking or resistance training [14] and may require specific skills and facility access, making it less accessible than other land-based activities [15].

Sport England’s Active Lives Adult Survey (2025) reported ∼4.2 million adults (aged 16+) swam at least twice in the past 28 days [17], with a further 5.6 million expressing an interest in swimming more [18]. However, this represents a decline of 658 000 regular swimmers over the past decade in comparison with increases in walking and other fitness or sporting activities over this period [17]. Given the national and global targets for PA, investment towards services promoting PA is urgently needed [11]. Understanding barriers and facilitators to PA amongst people with LTHCs [12] is key to shaping interventions, services and policy. However, there is currently little published UK data on swimming barriers in this group. The aim of this study was to analyse data from Swim England’s ‘England Swims’ survey to explore current perceptions of swimming and aquatic exercise.

## Methods

### Study design and participants

This cross-sectional study involved analysis of responses collected during the 2022 Swim England ‘England Swims’ survey (Supplementary Appendix SA). England Swims was a 4-week nationwide survey aimed at discovering the barriers preventing adults from accessing water-based PA. For this analysis, a subset of respondents from ethnically diverse communities were selected based on the following criteria: aged 16 or over and self-reported having a long-term (>12 months) physical or mental health condition that substantially affects their daily life. These individuals were drawn from the broader England Swims survey sample, which did not use LTHC status as a screening criterion.

### Survey development and content

The survey was co-developed by Swim England with a group of 'critical friends,' including individuals from ethnically diverse communities and organizations like the Muslim Sports Foundation and Sporting Equals, to ensure the list of barriers was comprehensive and culturally appropriate.

The questionnaire comprised 28 items exploring participants’ ethnic background, swimming engagement and ability, perceptions of swimming as an activity, and perceived barriers to participation. All individuals were eligible to respond regardless of their current swimming participation. Following the closed questions, all participants were invited to provide additional comments on barriers to swimming and factors that might support greater engagement in the activity.

### Survey process

The survey was promoted in May 2022 through a targeted marketing campaign using strategic partnerships with community organizations, online influencers, and digital advertising to reach ethnically diverse communities. The survey was delivered via SurveyMonkey, with response rates monitored daily to adjust outreach tactics. All personal information was handled securely in line with the General Data Protection Regulation and Data Protection Act (2018). By completing the survey, participants gave their consent for the information they provided to be used for the purpose specified within the information sheet.

### Ethical approval

This study was an evaluation using anonymized secondary data and does not require full research ethics review or favourable opinion for the purposes of publication. Instead, we completed a Data Privacy Impact Assessment (DPIA), under the direction of the University of Nottingham Information Compliance Unit. The research team were blinded to participant identity, maintaining anonymity throughout.

### Analysis

Demographic information was summarized using descriptive statistics. Participant responses were summarized descriptively. Free text responses were analysed thematically according to Miles and Huberman techniques of labelling, coding, categorizing, and theme development [19]. The process involved identifying commonalities in the data set and searching and comparing the free text responses to identify relationships and themes. Constant comparative techniques were used to ensure all perspectives were represented in the analysis, and deviant cases examined. The research team verified findings at each stage to ensure rigour and followed the STROBE reporting guidelines (Supplementary Appendix SB) in the production of this study.

### Research team

All three researchers involved in the analysis were white British. None had personal lived experience of LTHCs; however, one had experience as an informal carer to someone with a LTHC. Research Positionality.

The research team adopted a relativist ontology and an interpretivist epistemology. Descriptive analysis was conducted by LW and FM, and reflexivity was addressed through critical self-reflection and researcher consultation. Patient and public involvement (PPI).

PPI members with LTHCs were not involved in the design, or conduct, or reporting, or dissemination plans of this research.

### Equity, diversity, and inclusion statement

The study included participants from all socioeconomic levels, participants with different ethnicities and from minority groups. Methods of inclusion and data collection were the same for all participants. The author team consisted of two women and three men from different disciplines and with a wide range of experience in academia.

## Results

Of the 6608 individuals who opened the survey, 4615 responses were received (70% response rate). Of these, 816 (18%) reported a LTHC. Participants were predominantly white (*n* = 729), female (*n* = 468), and aged 42–57 (*n* = 333) with mental health (*n* = 463) the most prevalent LTHC (Table 1).

**Table 1.** Socio demographic data of all respondents with a LTHC (*n* = 816) and Swimming frequency of participants who had been swimming with the last year (*n* = 479)

	All respondents	
Respondent characteristics	No. of participants	Percentage (%)
**Gender**		
Man	312	38.2
Woman	468	57.4
Nonbinary	13	1.6
Transgender man	6	0.7
Transgender woman	1	0.1
Prefer to self-describe	6	0.7
Prefer not to say	10	1.2
**Age group**		
16–25	96	11.7
26–35	100	12.3
36–41	122	15
42–57	333	40.8
58–65	99	12.1
66–75	61	7.4
75+	2	0.3
Prefer not to say	3	0.4
**Ethnicity**		
White	729	89.3
Black	19	2.4
South Asian	25	3.1
East Asian	13	1.6
Mixed	19	2.3
other	11	1.3
**Disability or illness**		
Mental health	463	56.7
Long term pain	390	47.8
Mobility	385	47.2
Chronic health condition	302	37.0
Breathing	161	19.7
Dexterity	135	16.5
Memory	112	13.8
Hearing	92	11.3
Behavioural	83	10.2
Other	58	7.0
Learning	52	6.3
Speech	41	5.0
Prefer not to say	5	0.6

	**All respondents**	
	
**Swimming frequency**	**No. of participants**	**Percentage**

At least once a week	202	42.2
Two or three times and month	97	20.3
Once a month	33	6.9
Just once or twice in the last 12 months	144	30.1
I can’t remember	3	0.6

### Swimming ability

Of the 816 participants with a LTHC, 606 (74%) reported that they could swim, 212 (26%) reported they couldn’t.

### Swimming frequency

The majority, (*n* = 479, 59%) had been swimming within the last year, 312 (38%) last swam over 1 year ago and 25 (3%) have never been swimming. Swimming frequencies for those who had been swimming within the last year (*n* = 479) are reported in Table 1.

### Perceptions of swimming

Table 2 shows the perceptions of swimming amongst all participants.

**Table 2.** Thinking about swimming, do you agree or disagree that swimming is an activity that is:

	Agree	Disagree	I don’t know
Enjoyable and satisfying	714	44	59
Important life skill to have	808	5	4
Safe	680	81	55
Open to all	527	221	68
Only for particular groups	148	571	96
Relevant to me	643	56	118
Accessible	363	333	121
Competitive	437	247	132
Easy to take part in	442	309	65
Social	523	197	97
A fun family activity	720	33	63

### Barriers

Table 3 presents the prevalence of common barriers to swimming.

**Table 3.** Most common barriers to swimming amongst non-swimmers (*n* = 484)

Barrier	Number of participants	Percentage (%)
I have a lack of disposable income	208	43.0
It is too expensive	191	39.5
I lack motivation as I’m not satisfied with my life these days	178	36.8
The swimming sessions I like aren’t at convenient times	159	32.9
I lack motivation as I feel lonely or isolated most of the time	147	30.4
I have no-one to go with	143	29.5

### Analysis of free text responses

Four themes related to swimming were identified with supporting free test responses and respondent characteristics reported (Supplementary Appendix SC).

- Confidence
- Fear of discrimination, stigma or unwanted attention
- Availability
- (Un)welcoming environment

#### Confidence

Confidence-related barriers to swimming were frequently reported. Water confidence was understandably a key consideration, with swimming ability frequently mentioned. For those unable to swim (or with limited ability), the prospect of entering the pool was intimidating for fear of ‘looking stupid’ especially in comparison to more able swimmers:

*‘I can’t swim so would look stupid dipping my toes in’ (R242)*

Shared lane swimming was also problematic. Lanes were often perceived as overcrowded making less confident swimmers, or those with a physical disability, feel vulnerable:

*‘Competitive swimmers should have own time/lane. I am not confident in water but can swim. Competitive swimmers are impatient and overtake, making me afraid of drowning’ (R362)*

*‘I am physically disabled, and I would like sessions where I had a lane to myself so I’m not worried about people bumping into me and hurting me’ (R619)*

Some participants were fearful of water or of falling on the poolside with many reporting unfortunate past experiences. These influenced their perceptions of swimming:

*‘My children had a bad experience of drowning but luckily they have been rescued by guides, after that, I am scared of go swimming and so are my kids’ (R392)*

To mitigate these concerns, some described the need for carers or some form of social support to give them the confidence and impetus to return to the pool:

*‘Sometimes swimming can get lonely so that shatters my motivation to keep doing it*’ *(R718)*

*‘Need someone to go with me. Am nervous of falling around the pool’ (R137)*

For those who had experienced health issues, returning to swimming was considered challenging. Participants described how their experience (e.g. COVID, cancer, surgery, menopause) had adversely affected their confidence.

*‘I used to go swimming once a week before the Covid pandemic… I now lack the confidence to return as I’m still worried about catching Covid’ (R345)*

#### Fear of discrimination, stigma, or unwanted attention

A number of participants associated swimming and aquatic exercise with adverse social experiences. The phrases ‘self-conscious’ and ‘body shame’ were frequently expressed. Common causes of concern were body weight and skin conditions. The need to undress and wear swim clothing further compounded individuals’ insecurities.

‘*I’m obese, disabled and use walking aids… I hate how I look, and don’t feel comfortable in very little clothing or getting to and from the pool’ (R580)*

*‘I have a visible skin condition and fear that people will judge, and I will be made to feel uncomfortable’ (R54)*

Participants also described poor experiences whilst in the pool and called into question the support that might be available to them:

‘*I feel that I’m judged for being slow and I’m in the way, and that if I did need help, no one would know how to help me’ (R580)*

*‘As a fat person I don’t feel welcome in swimming pools…. I don’t believe that instructors or lifeguards would try to help me if I was having difficulties… I loved swimming when I was a kid, but as an adult, I’ve only had poor experiences’ (R586)*

Lack of cultural humility and discrimination in the aquatic environment had also been experienced by participants. Many perceived that the pools did not cater for their faith requirements and lacked an understanding of their cultural and religious beliefs to the extent that it stopped aquatic participation. Many of the frustrations focused on inclusive swimming sessions (female only) and the provision of culturally sensitive changing facilities:

*‘The local pool isn’t inclusive. They have repeatedly ignored or made excuses when asked to provide inclusive swimming sessions with female lifeguards. We have been requesting their assistance for over 6 years to no avail’ (R418)*

*‘Often changing rooms are communal and Muslim men and women are not allowed to take their clothes off in front of others even if of the same sex. I have to miss 10 [minutes] of the session so I can change in the shower cubicle to avoid the queue as all the other Muslim ladies have to do the same as we cannot use the communal changing rooms’ (R397)*

Similar discrimination was described by individuals who identified as non-cis. Experiences ranged from feeling uncomfortable in the aquatic environment, through to direct harassment:

*‘I’m trans and early in my transition—it’s hard to navigate changing rooms without scaring/bothering/upsetting others’ (R17)*

*‘I have experienced transphobic harassment from other swimmers and pool staff in the past’ (R543)*

#### Availability

Participants repeatedly emphasized the lack of alignment between swimming session schedules and personal commitments, such as employment, and childcare. Many reported difficulties in accessing public swimming sessions due to restrictive scheduling and the long-term impact of COVID:

*‘There are no public swimming sessions that I can just turn up to… I used to enjoy swimming straight after work, but since the pandemic, the local pools don’t open for a public swim at these times’ (R201)*

Many participants expressed frustration with pool operators’ focus on members-only swimming clubs, which limit access for recreational swimming:

*‘Pool operators do not value recreational swimmers enough. Evening programs focus on clubs and Learn to Swim. Some people requiring low-impact activities are not old and work during the daytime so evening access is very important’ (R648)*

The availability barrier extended further than pool scheduling. Participants reported local pool closures, which increased the logistical and financial burden of accessing a pool:

*‘My local pool closed down and hasn’t reopened therefore to travel to my nearest pool would prove too expensive and time consuming’ (R124)*

Financial constraints emerged as a significant deterrent, highlighting cost as a prohibitive factor preventing regular swimming participation.

*‘I’m on a low income but am not eligible for any benefits. All my money goes on the bills… so swimming is too expensive’ (R420)*

*‘Cost of taking a family each time is £10 a week or £40 a month minimum. Family monthly passes need to be introduced’ (R295)*

The desire for open water or wild swimming provision emerged throughout the study. However, several participants reported the lack of safe outdoor swimming facilities nearby.

*‘I would like to swim outdoors more, in a safe space that is close by, but all the lidos have closed’ (R457)*

#### (Un)welcoming environment

The physical and social conditions of facilities were key deterrents. Many participants commented on safety concerns related to mixed changing facilities.

*‘Women who are survivors of sexual violence and women from religious communities are being driven away from swimming by the absolute failure to recognise our needs to single sex services…’ (R651)*

*‘It is important for women to feel safe both physically and socially. A pool and changing room is a place where women and girls can feel particularly vulnerable, due to actual or near nakedness’ (R653)*

Many participants expressed a desire for female-only swim sessions, citing cultural and religious reasons. While some pools offer such sessions, many felt they didn’t meet their needs.

*‘I’m a Muslim woman and the ladies only sessions are either not at suitable times or too crowded and they don’t cater for women who can actually swim’ (R281)*

*‘Lack of male only and women only sessions with the corresponding same sex lifeguard in a clean not overlooked pool without members of the public or staff being able to walk in’ (R397)*

Participants often expressed concerns regarding the cleanliness of swimming facilities and water quality. Many individuals reported a perceived lack of proper maintenance and investment, expressing frustration with the outdated, dilapidated, and damaged facilities, while highlighting various issues that posed hygiene and safety concerns for pool users.

*‘Pool is run down, water too cold, closed too often, changing rooms are revolting… as are the other facilities- mould mildew, holes, broken and smell and toilets are even more disgusting and rarely work, pool overcrowded’ (R378)*

*‘Disabled changing rooms are not safe to use’ (R260)*

The theme of water quality transcended into other issues, including water temperature. Many stated that the water was simply too cold. Those with a health-issue or disability reported the negative impacts of low pool temperatures and noted the exacerbation of their symptoms or the discomfort they suffered:

‘*Getting too cold in the water as I have fibromyalgia’ (R43)*

*‘I have CES [cauda equina syndrome] and worry about the temperature of the pool in case by back goes into spasms if the water is too cold’ (R85)*

*‘I have anorexia, and I get cold really easily and being in water makes me really cold’ (R165)*

A common concern was high chlorine levels in indoor pools, causing respiratory, eye, and skin irritation.

*‘The pools are too chlorinated. If it was at lower levels, I would be able to go for an hour or so but as it stands, I can’t even breathe walking past’ (R589)*

Several participants raised concerns about noise pollution and safety in the aquatic setting. It was apparent that overcrowding was a significant issue which prevented many individuals from participating in swimming:

*‘The local swimming pool does not offer quiet times for family swimming for those with autism (*e.g. *SEN [special educational needs] sessions). Most family times are too busy and too noisy’ (R257)*

Lack of disability awareness and training amongst pool staff was identified as an issue. Individuals felt that they could not approach staff and were forgotten about. Communication with the pool operators and availability of advice or specially trained staff, especially for those with a disability was an evident concern:

*‘Lack of disability awareness among staff, lack of understanding of autism among staff, lack of physical access, lack of information about accessibility, disabled people frequently get forgotten about and left out of conversations’ (R539)*

*‘Local pools won’t let me swim due to my mobility/health issues due to not having enough lifeguards or I am not a strong enough swimmer to be in the pool (I can swim at least 2 or 3 lengths without stopping at the moment)’ (R121)*

## Discussion

Recent data show a decline in regular swimming and aquatic exercise over the past decade, with numbers falling during the COVID-19 pandemic and not returning to pre-pandemic levels [17]. The results of this study provide insights into perceptions of and barriers to these activities amongst individuals with LTHCs. Our analysis reveals a complex combination of personal, social and environmental factors that influence swimming and aquatic exercise participation. These findings align with recent research on PA barriers in LTHCs [12] and the ecological model of health behaviour (Fig. 1) which emphasises that health behaviours are influenced by multiple, interacting levels. Additionally, these results highlight the intersectionality of LTHCs with other protected characteristics, adding specific insights to the aquatic context which can educate and inform healthcare professionals, leisure providers and other stakeholders interested in this area.

Lack of confidence emerged as a significant barrier, particularly for individuals with limited swimming ability or those returning to swimming after health issues. Swimming ability was highlighted, with one in four participants reporting that they cannot swim. This finding is consistent with previous research identifying self-efficacy as a critical determinant of PA participation [20]. The COVID-19 pandemic exacerbated this, creating hesitancy to return and highlighting the need for targeted interventions to rebuild water confidence in vulnerable individuals.

Body image concerns and fear of discrimination were prevalent, reflecting broader societal issues related to body stigma and inclusion [21]. Participants reported feeling self-conscious about their bodies, particularly in the exposed environment of a swimming pool. Body image concerns are known barriers to PA, especially in activities that require minimal clothing [22, 23]. The aquatic environment presents unique challenges, as swimwear offers less coverage than typical exercise attire. For those with visible LTHCs this issue is exacerbated, with intersectionality becoming apparent.

Cultural and religious considerations were prominent, participants expressed frustration at the lack of appropriate accommodation for their needs. Highlighting significant gaps in service provision that may disproportionately affect certain communities. Previous research has identified that individuals from ethnically diverse backgrounds face additional barriers to PA participation including higher burden of disease and cultural expectations including preservation of modesty (single-sex sessions, religious clothing) [24]. Our findings suggest that aquatic leisure providers may not be adequately addressing these needs regarding changing facilities, swimming session scheduling, staff resourcing and appropriate staff training.

Scheduling and availability issues represent systemic barriers that impact water-based PA participation. The reported focus on swimming clubs and lessons at the expense of recreational swimming sessions suggests a misalignment between pool programming and the needs of individuals with LTHCs who may benefit from regular water-based activity [25]. Pool closures and prohibitive costs were also reported as significant barriers, potentially disproportionately affecting those with LTHCs, who may have additional constraints on their time and financial resources [26].

The perception of swimming facilities as unwelcoming environments was a recurring theme, with participants reporting concerns about cleanliness, maintenance and safety. Low water temperature was specifically highlighted as a barrier for those with certain health conditions such as fibromyalgia or anorexia. Warmer water temperatures can enhance comfort and may have therapeutic benefits for certain health conditions [13]. Some participants reported issues with chlorinated water due to reactions to the chemical and therefore highlighted their need for open-water or freshwater swimming options. However, participants reported that these are rare, highlighting a service provision that should be considered.

## Research/policy implications

Despite the barriers highlighted, most participants (99%) reported that they felt swimming was an important life skill and 88% of participants considered it enjoyable and satisfying. Together this suggests that to achieve Swim England’s goal of increasing participation, a multi-faceted approach is needed to promote water-based PA at a time when nationwide figures suggest lower participation than previously [17]. Given the health benefits associated with swimming for individuals with LTHCs, research examining the cost-effectiveness of infrastructure investment and programming changes would be valuable for informing policy decisions.

## Limitations

As a cross-sectional survey relying on self-reported data, findings may be subject to recall and response biases. The lack of formal questionnaire validation may impact reliability and comparability, although ‘critical friends’ were involved to enhance cultural relevance and inclusivity. The open access nature of the survey, combined with a recruitment strategy that targeted both swimming and non-swimming communities may have led to over representing individuals with particularly strong views or barriers to swimming.

## Conclusion

This study provides important insights into current perceptions of and the barriers to swimming and aquatic exercise participation among individuals with LTHCs in England. The findings reveal that despite generally positive perceptions of swimming, multiple barriers prevent many from engaging. Targeted interventions to make swimming more accessible and appealing to individuals with LTHCs are needed. These could include adult swimming programmes, staff training on disability awareness and cultural sensitivity, more flexible scheduling options, and improvements to facility design and maintenance. Attention should be paid to creating inclusive environments that respect cultural and religious needs and accommodate various health conditions. Future research should explore the effectiveness of specific interventions aimed at addressing these barriers, as well as investigating the experiences of subgroups of individuals with LTHCs who may face unique challenges related to their condition. Pool operators face significant financial and operational challenges around programming and facility upkeep; therefore, future research should also explore cost-effectiveness and best practices for supporting individuals with LTHC participating in aquatic activity.

## Supplementary Material

ckag075_Supplementary_Data

## Data Availability

The datasets used and/or analysed during this study are available from the corresponding author on reasonable request.

## Patient and public involvement

Patients and/or the public were not involved in the design, or conduct, or reporting, or dissemination plans of this research.

Key points

- Swimming and aquatic exercise benefit people with long-term health conditions, yet participation remains low due to multiple barriers.
- This study provides the first large-scale UK-wide data on experiences of swimming among people with LTHCs.
- Environmental and interpersonal factors strongly shape willingness to engage in aquatic exercise.
- There is a need for inclusive, co-designed interventions and policies that reduce financial, environmental, and social barriers.
