# Supplementary material for: Exploring experiences of swimming and aquatic exercise in individuals with long term health conditions: results from Swim England’s ‘England Swims’ survey
Source: Eur J Public Health. 2026 Jul 10;36(4):ckag075. doi: 10.1093/eurpub/ckag075 (PMC13349663; doi:10.1093/eurpub/ckag075)

Supplementary file S1

**Swim England – England Swims Survey**

**About the survey**

The survey was created by Swim England with the support from Muslim Sports Foundation and Sporting Equals to ensure the question responses were appropriate and inclusive for ethnically diverse communities and those from different faith groups.

The survey was distributed with a snowball sampling approach. It was shared with a number of Swim England partners, to share via their email distribution lists, newsletters, social media etc. These included NHS England, pool operators, sport sector bodies (such as Sporting Equals, Muslim Sports Foundation, Activity Alliance), and via Swim England’s own channels. Swim England also reached out to a variety of faith and ethnicity groups to ask for them to promote to their membership. In addition, Sporting Equals used their field team of ‘activators’ to take the survey into ethnically diverse communities, where survey completion was encouraged.

The aim was to reach as many non-swimming individuals as possible, with focused efforts on ethnically diverse communities. The sample was weighted against gender, age and ethnicity to ensure it was representative of England population. The LTHC dataset (filtering only those who had a LTHC) was not weighted.

The full questionnaire is provided below. It was distributed online using survey monkey, but the attached version was also printed and shared with communities (face to face), to support completion by those not comfortable with technology, or needing support with translation.

Swim England have published a headline findings pack on the Swim England website, which can be downloaded for free. This covers all 4,615 responses. In addition, a number of ‘Spotlight on…’ findings packs were created for community groups, such as South Asian community, Muslim community, LTHC community. These are also available from the [same webpage](https://www.swimming.org/swimengland/england-swims-research-findings/).To date there are no other publications planned, and there have been no academic publications using the England Swims dataset except for this one.

Continue to the next page for survey.


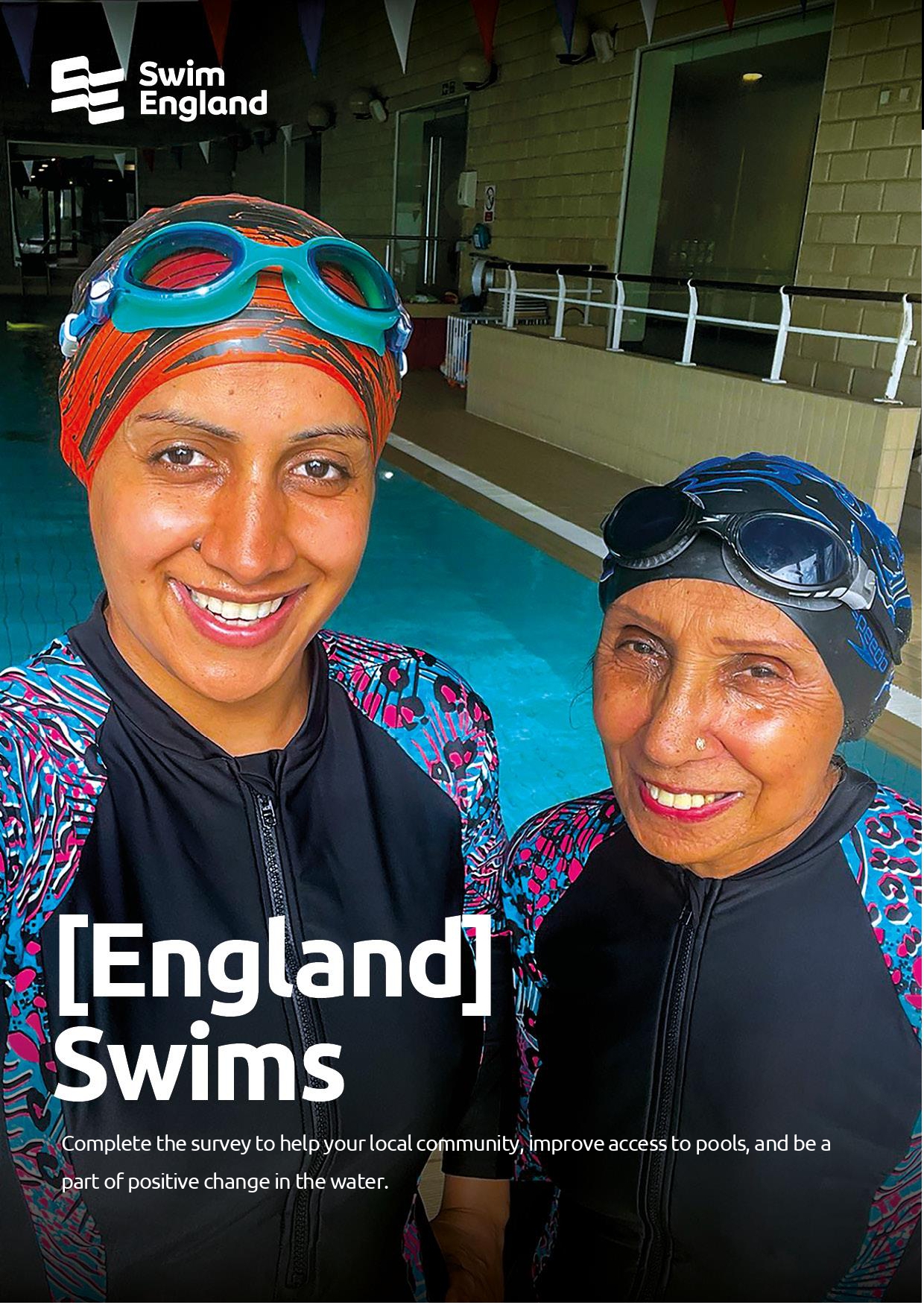

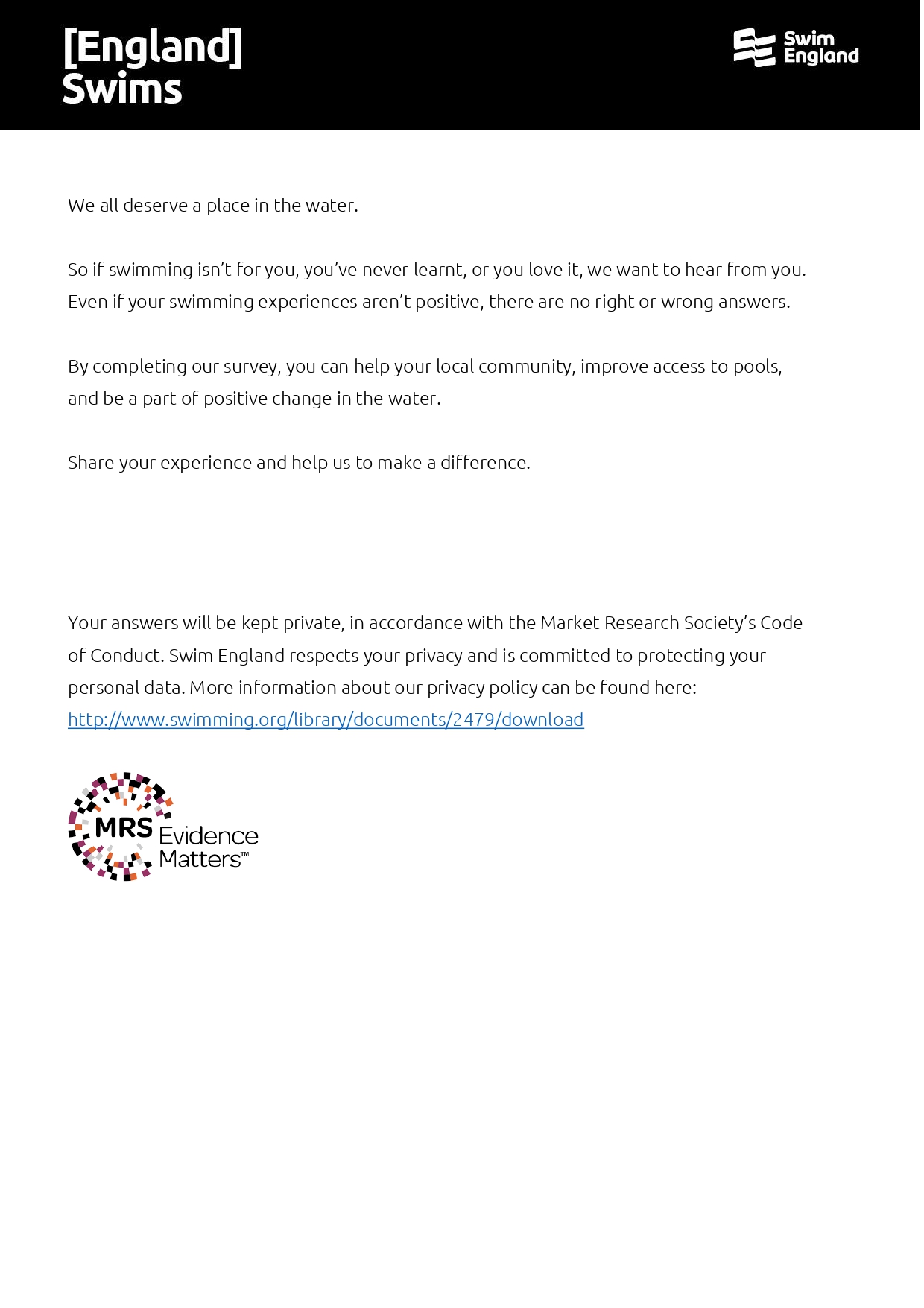

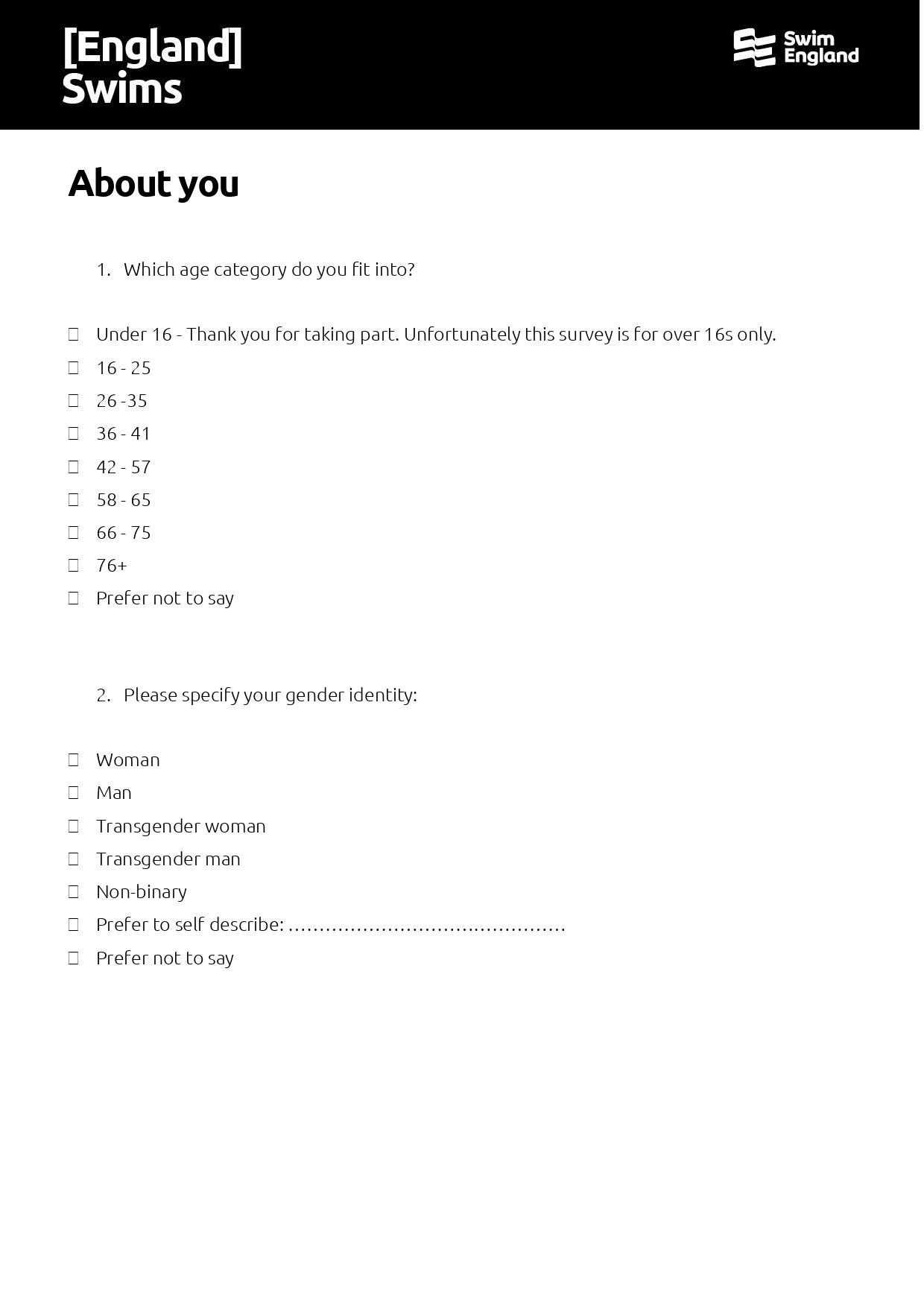

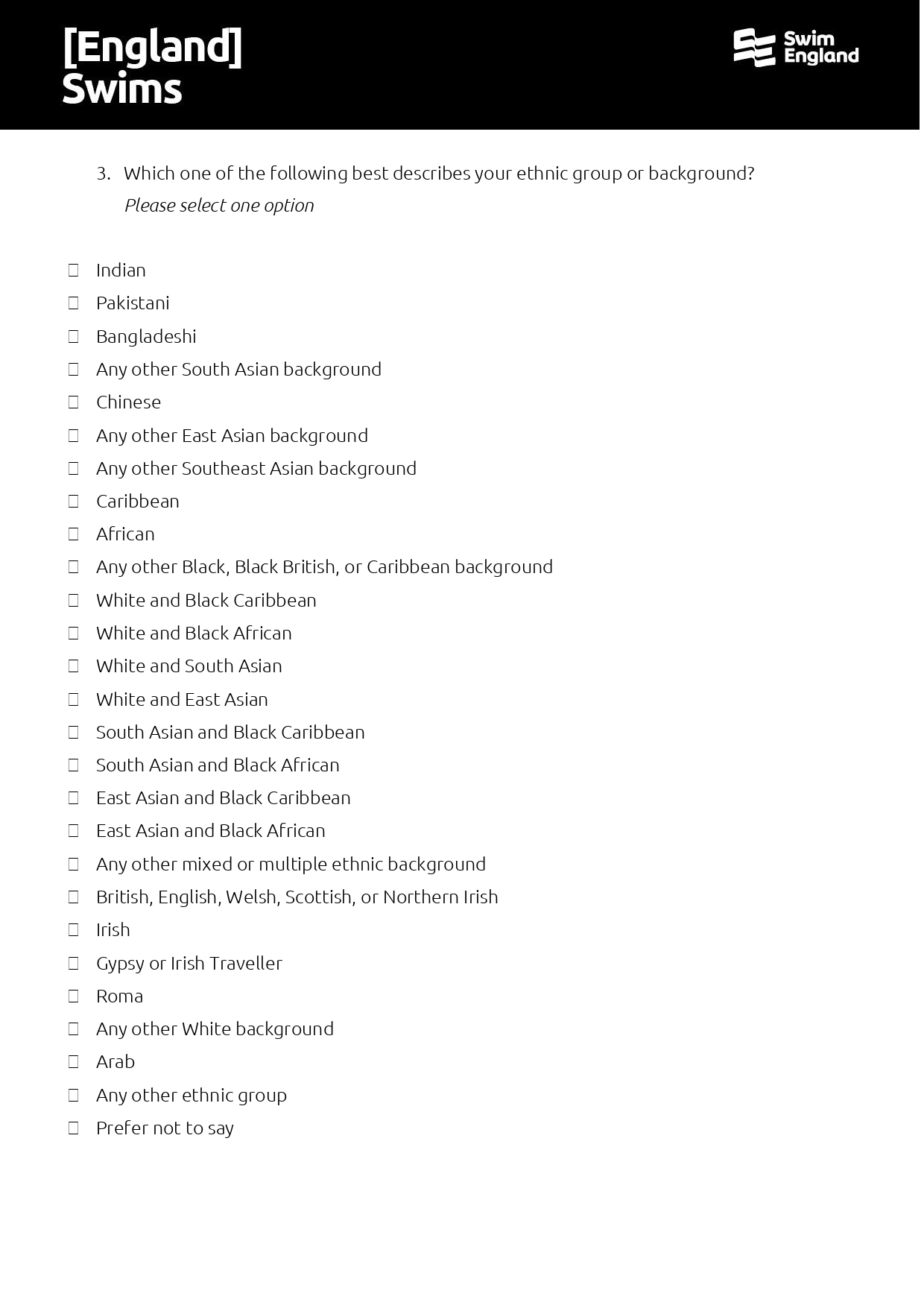

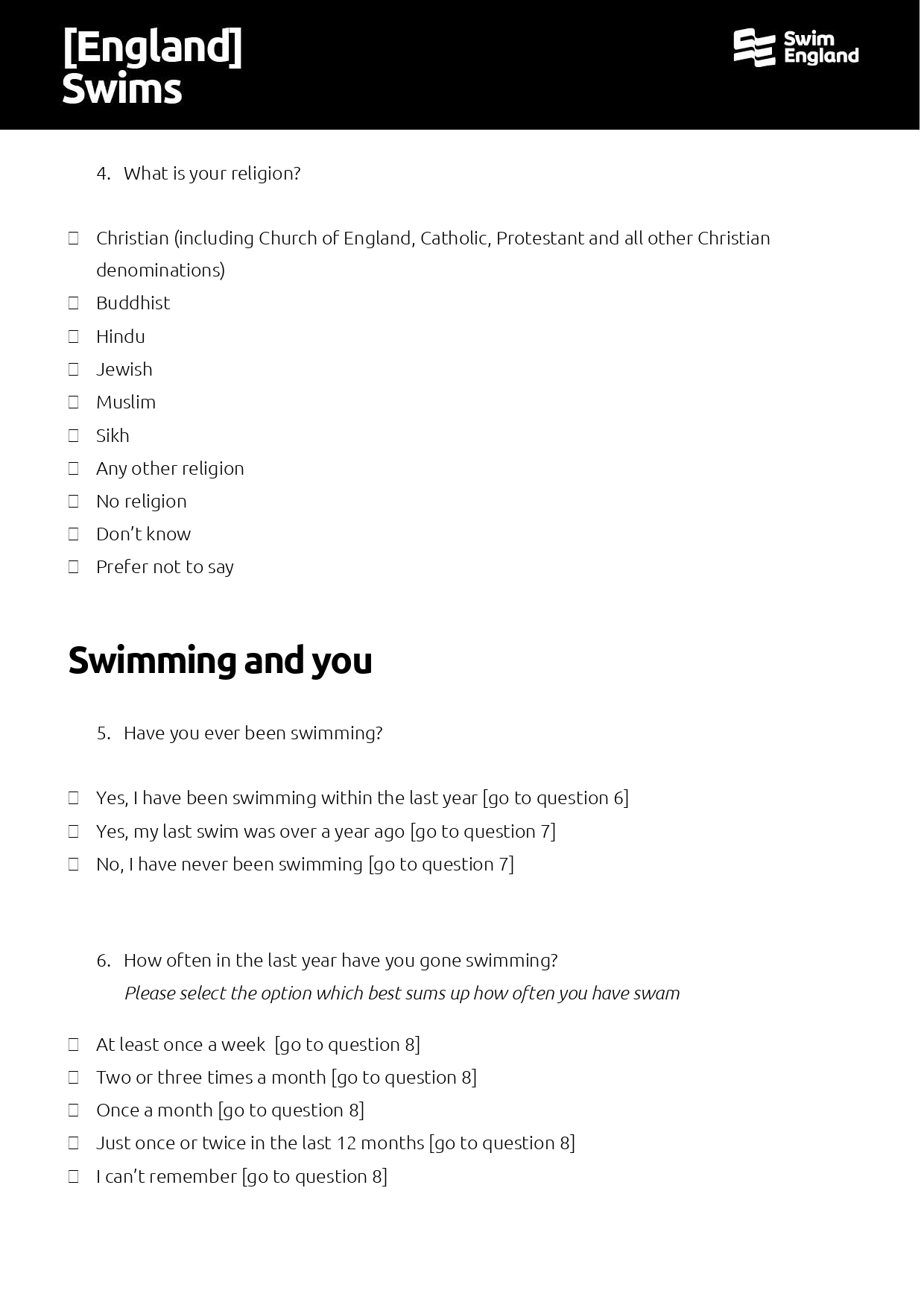

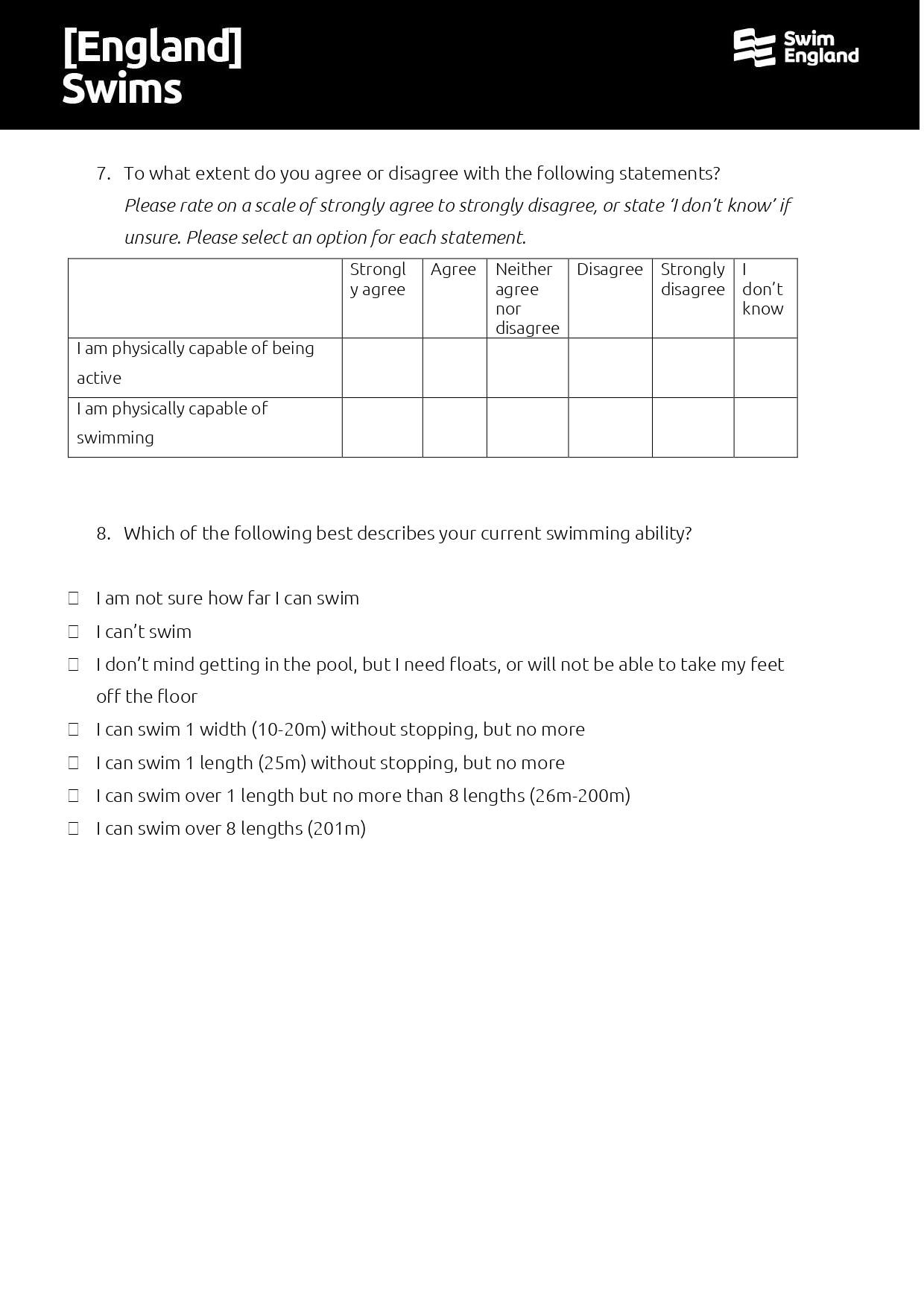

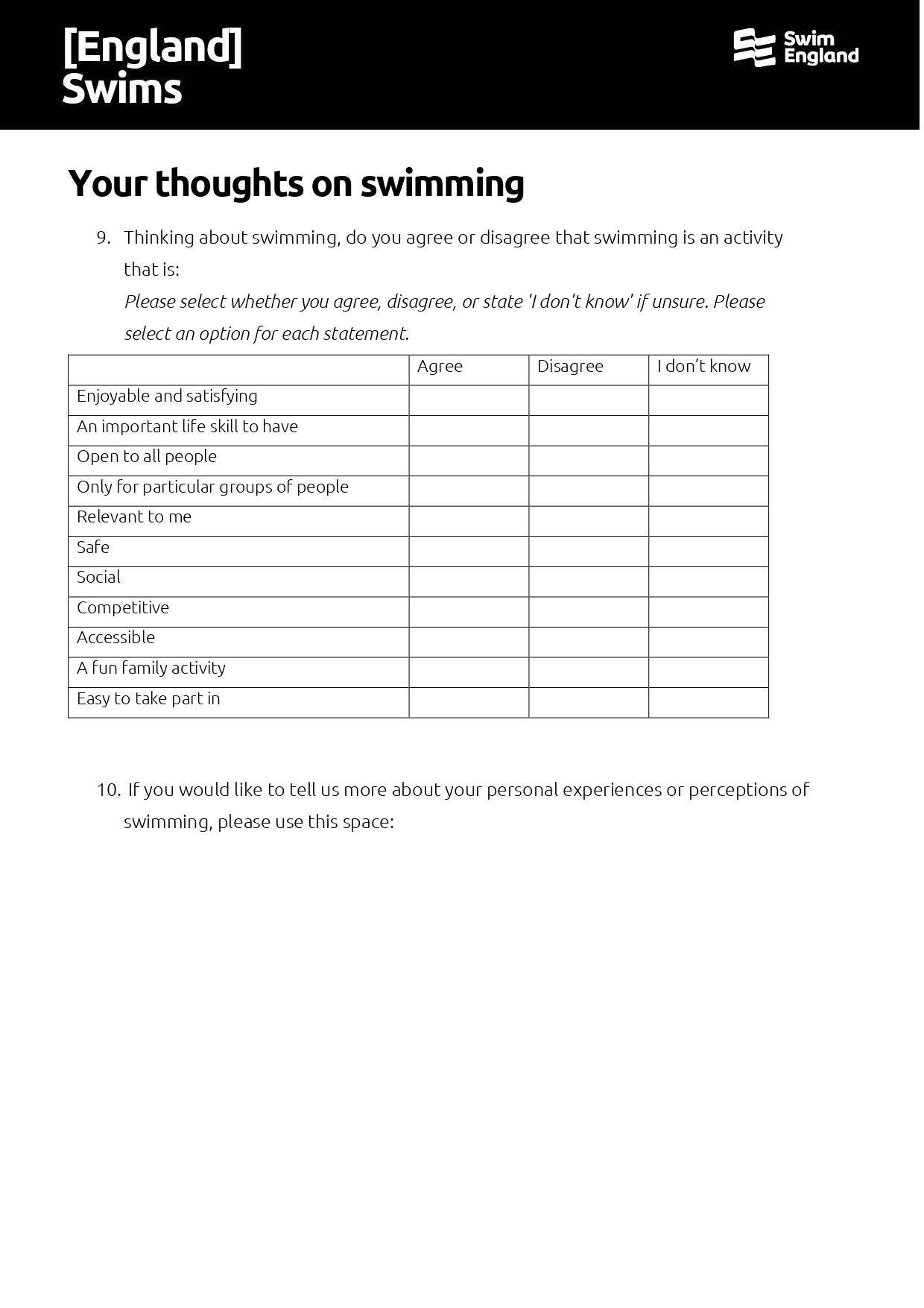

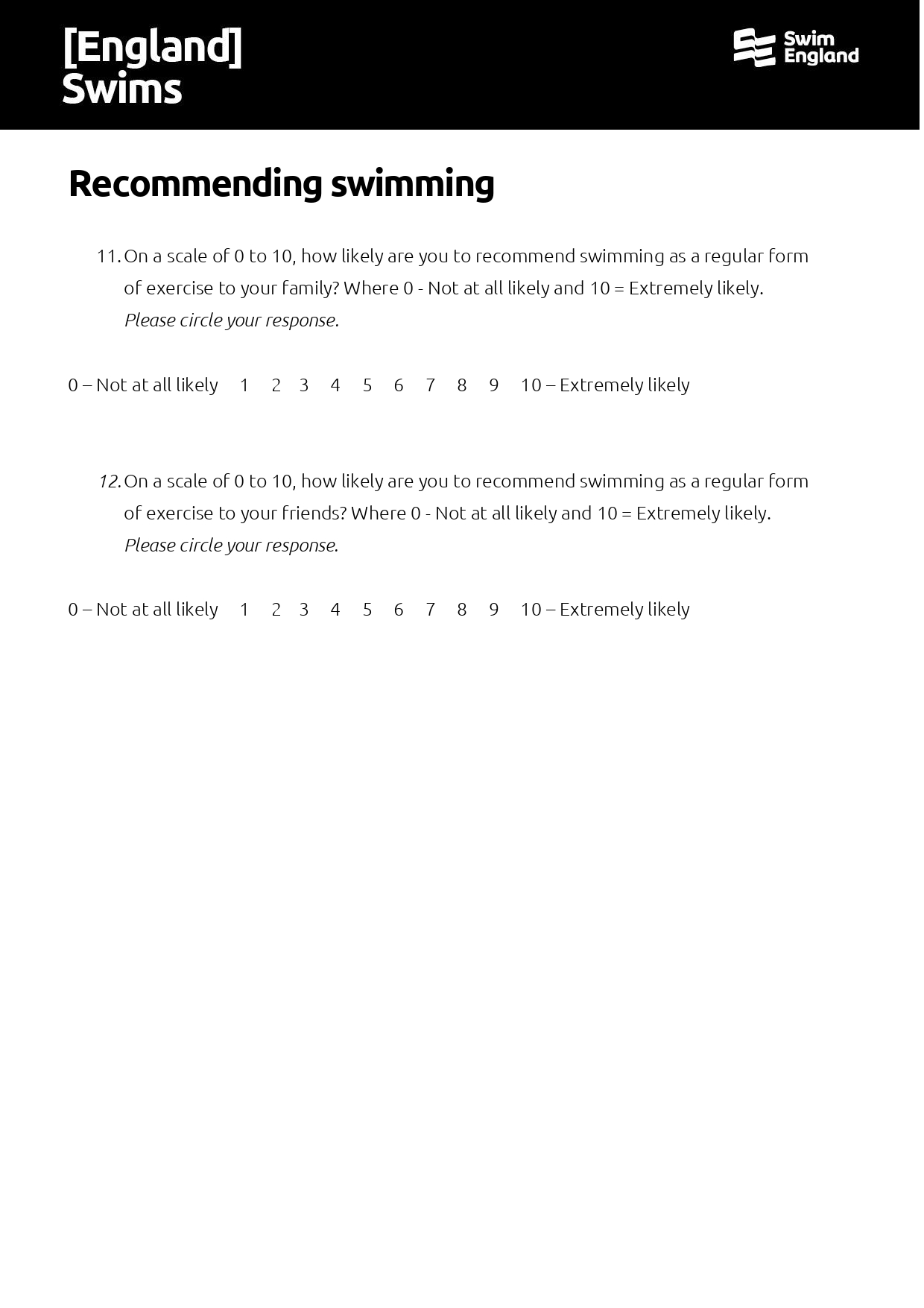

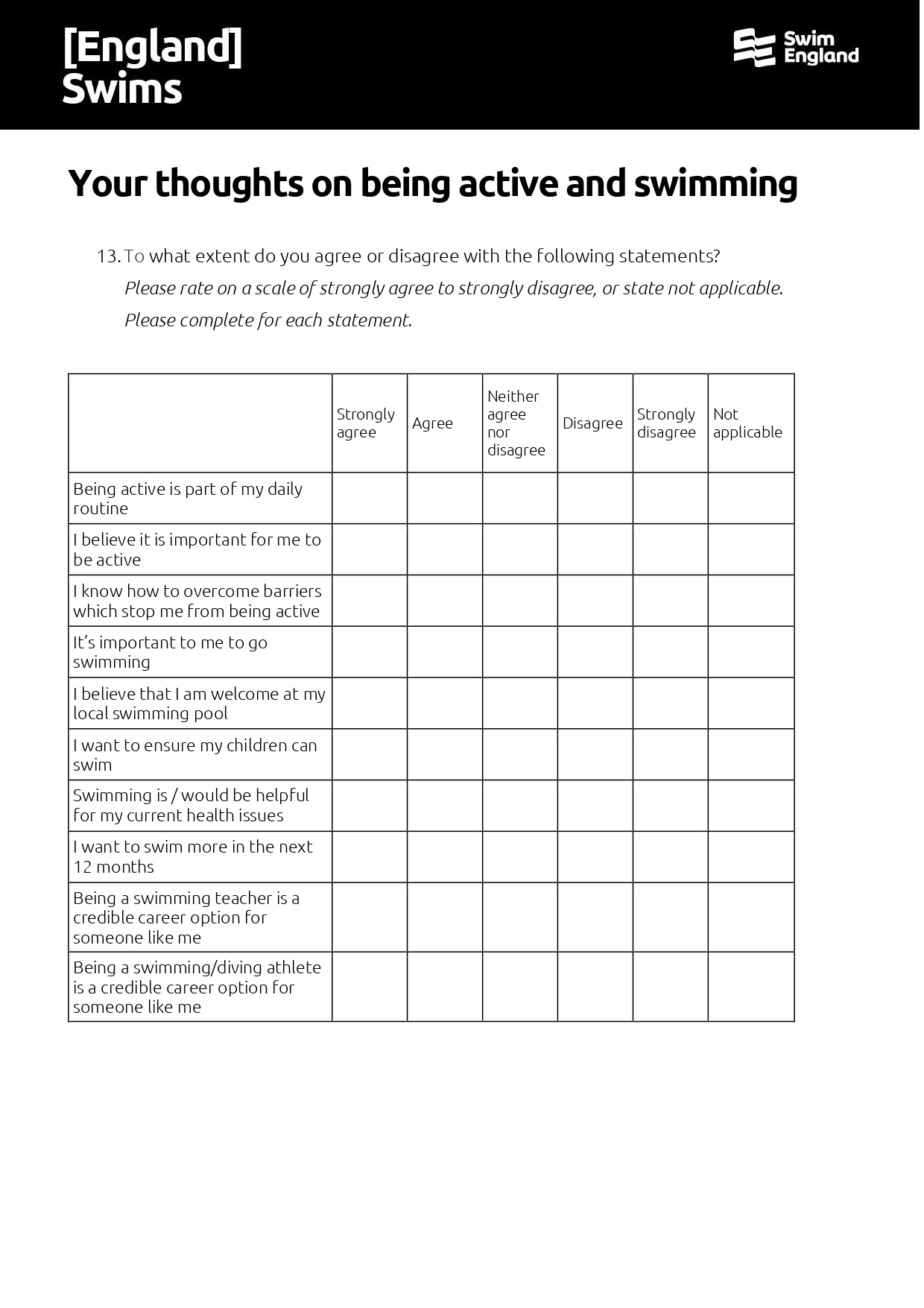

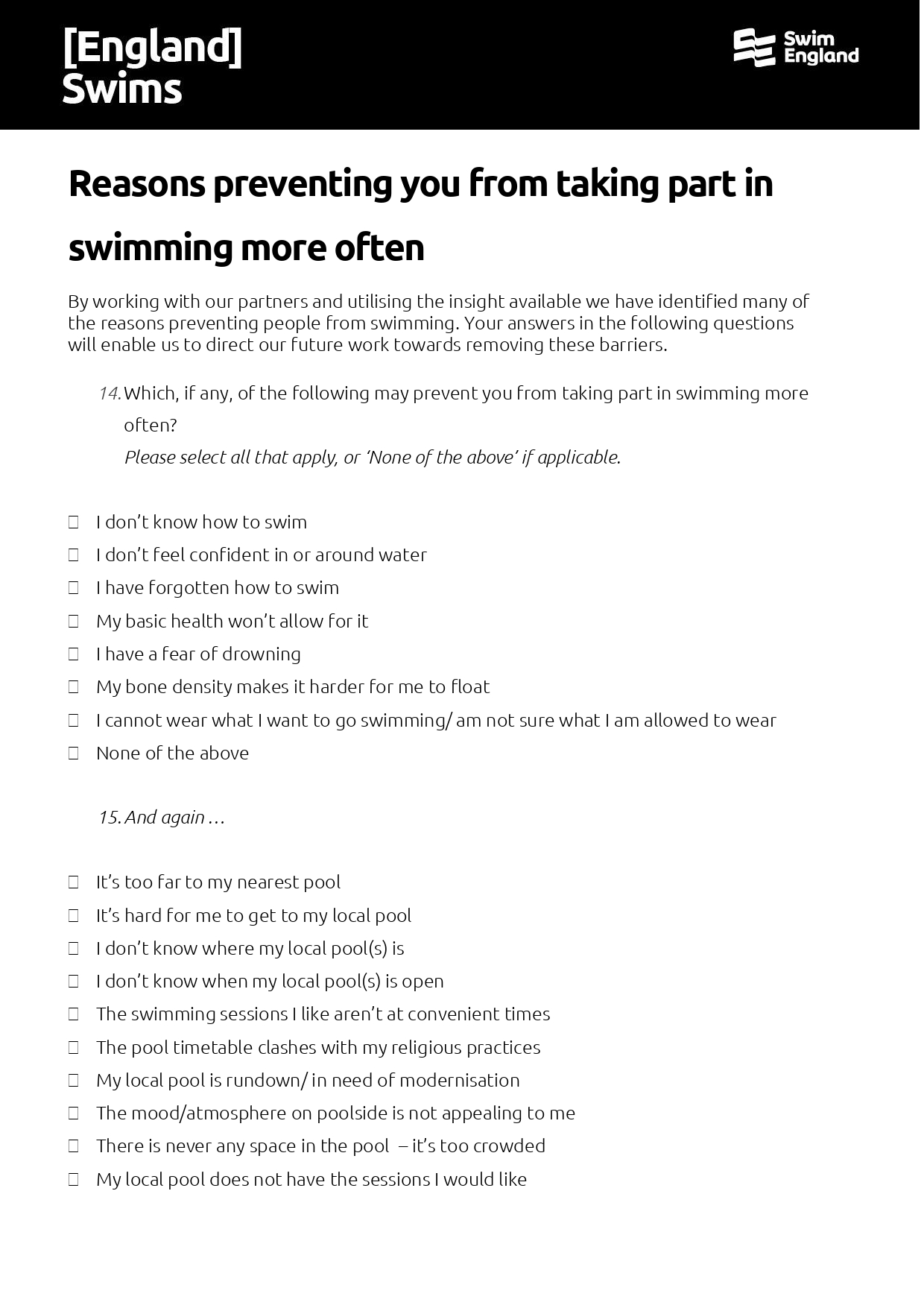

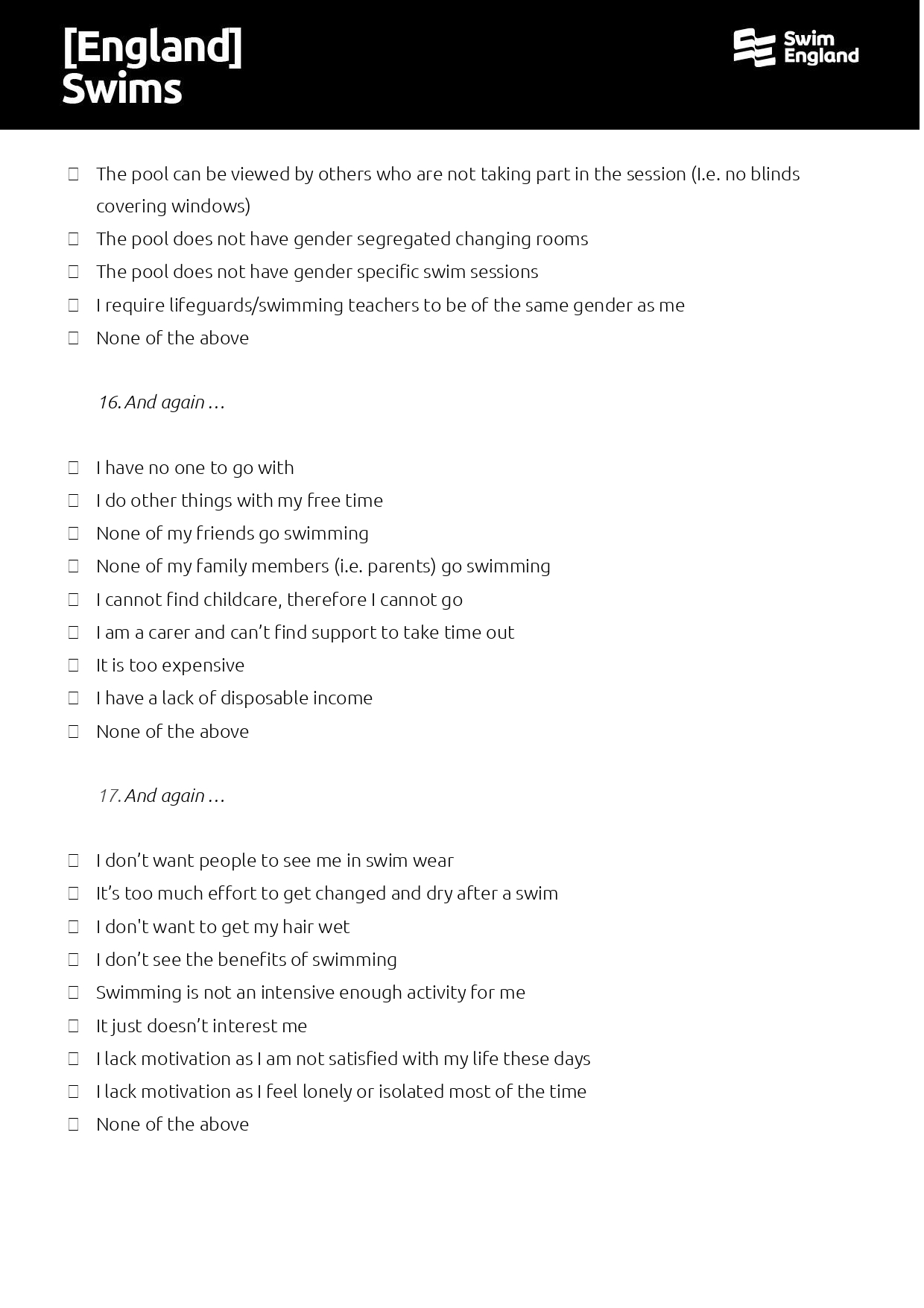

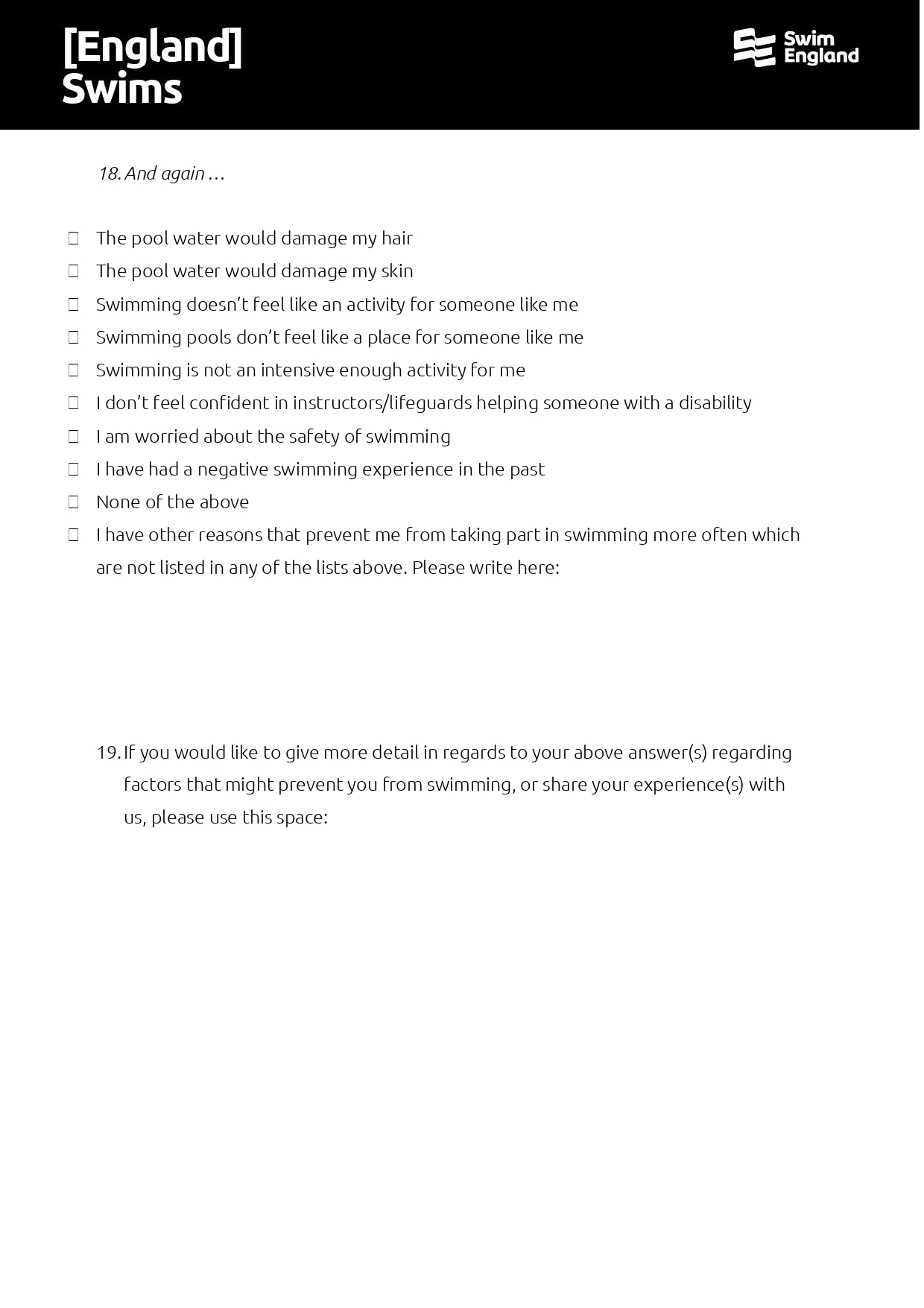

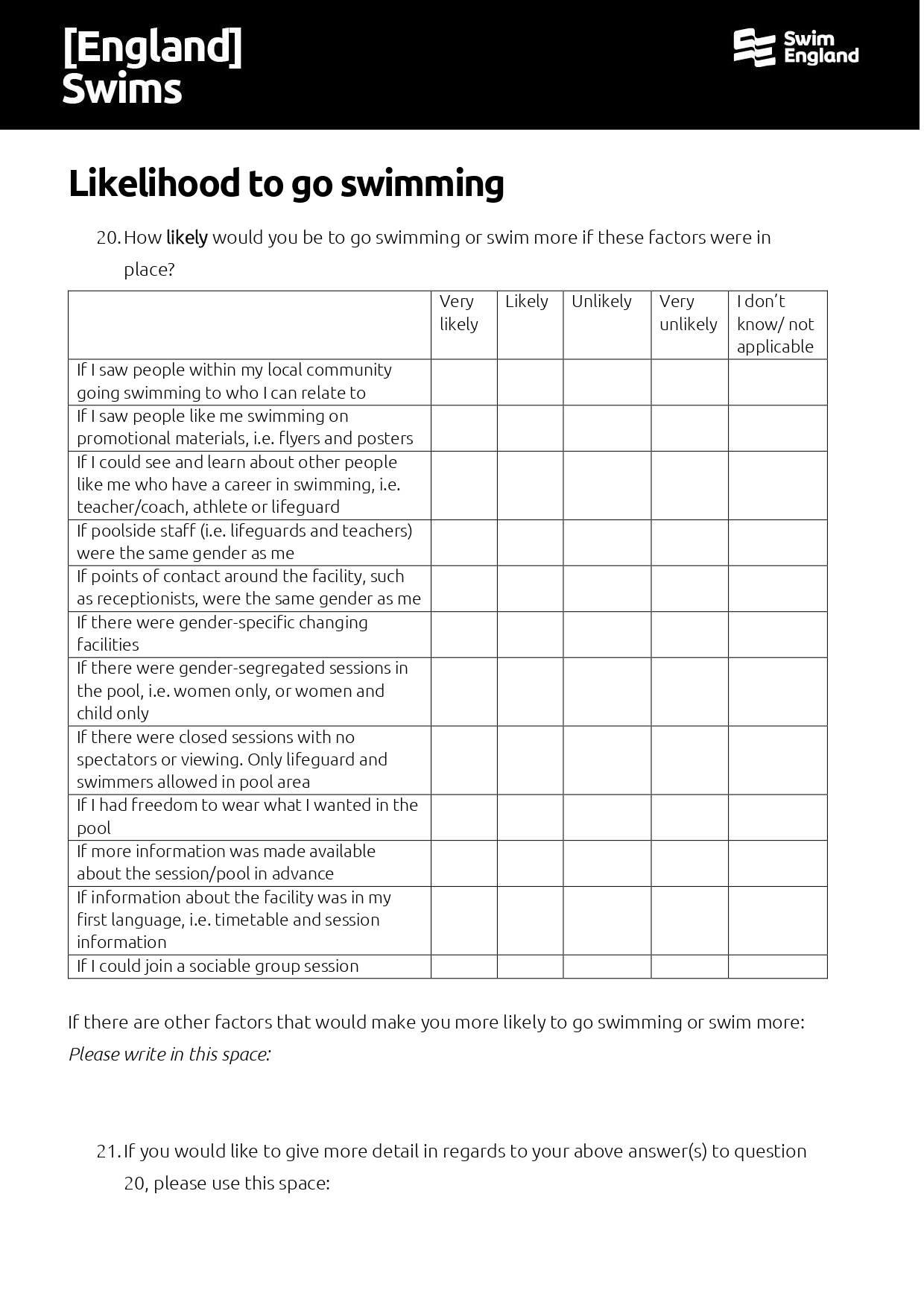

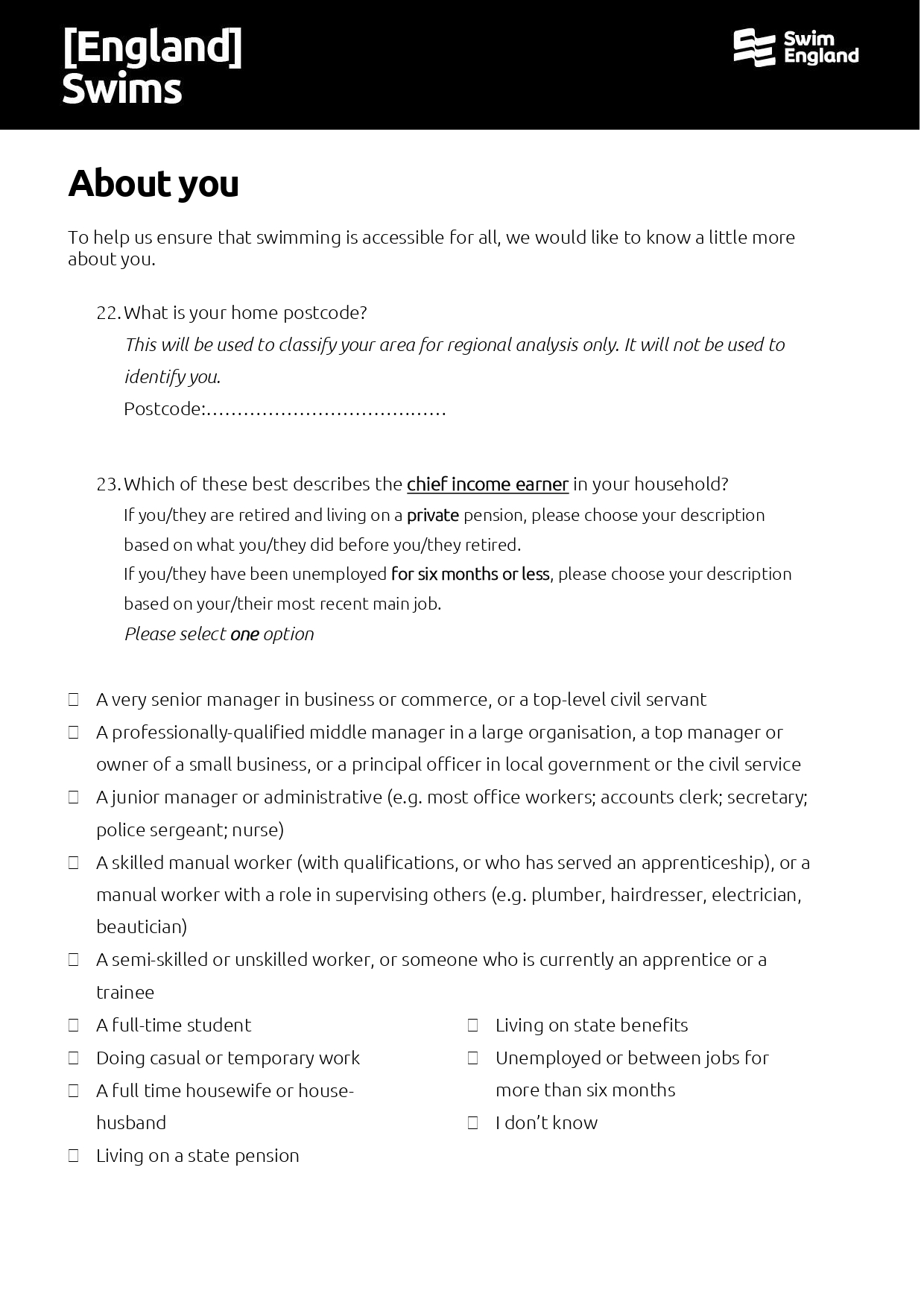

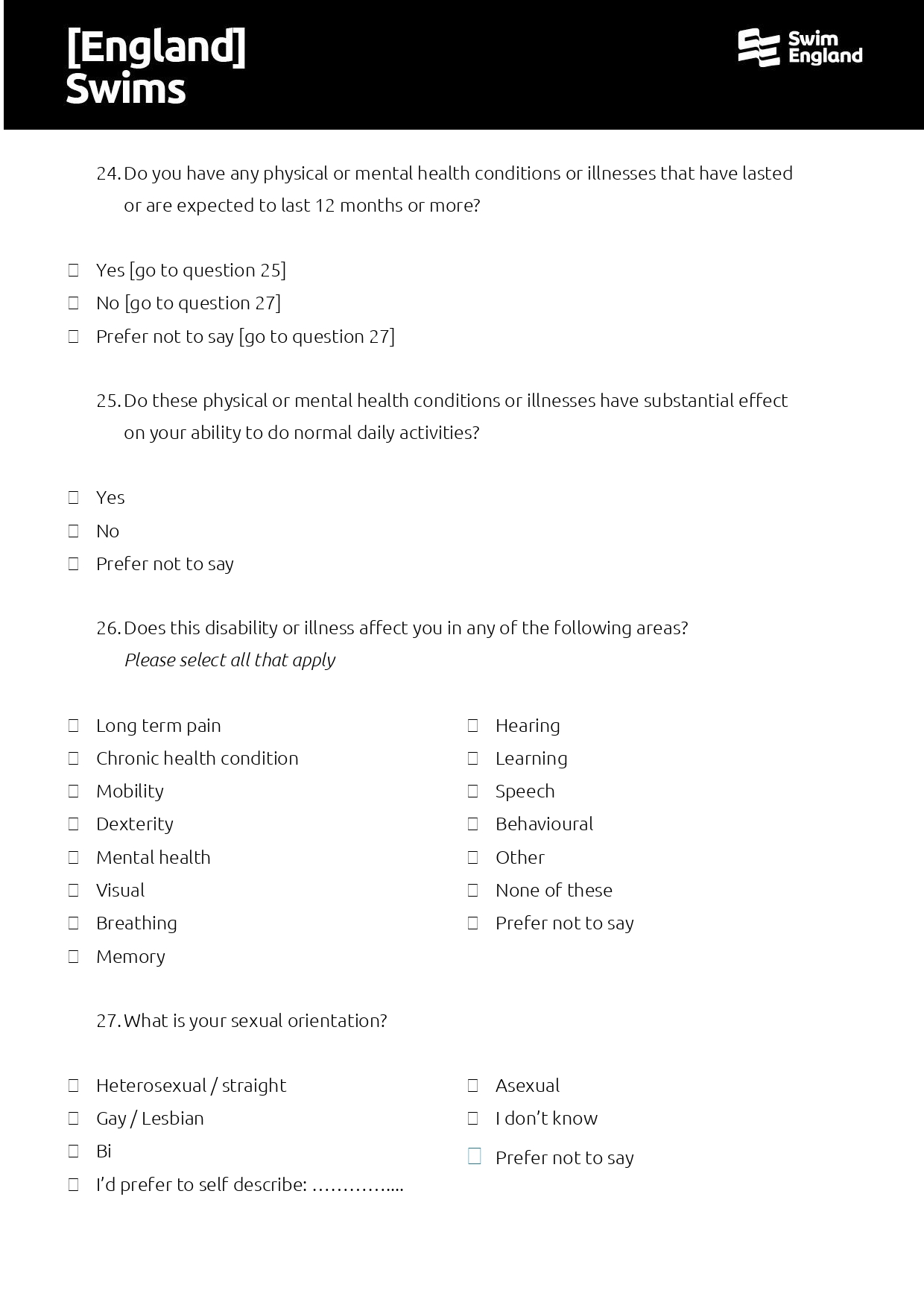

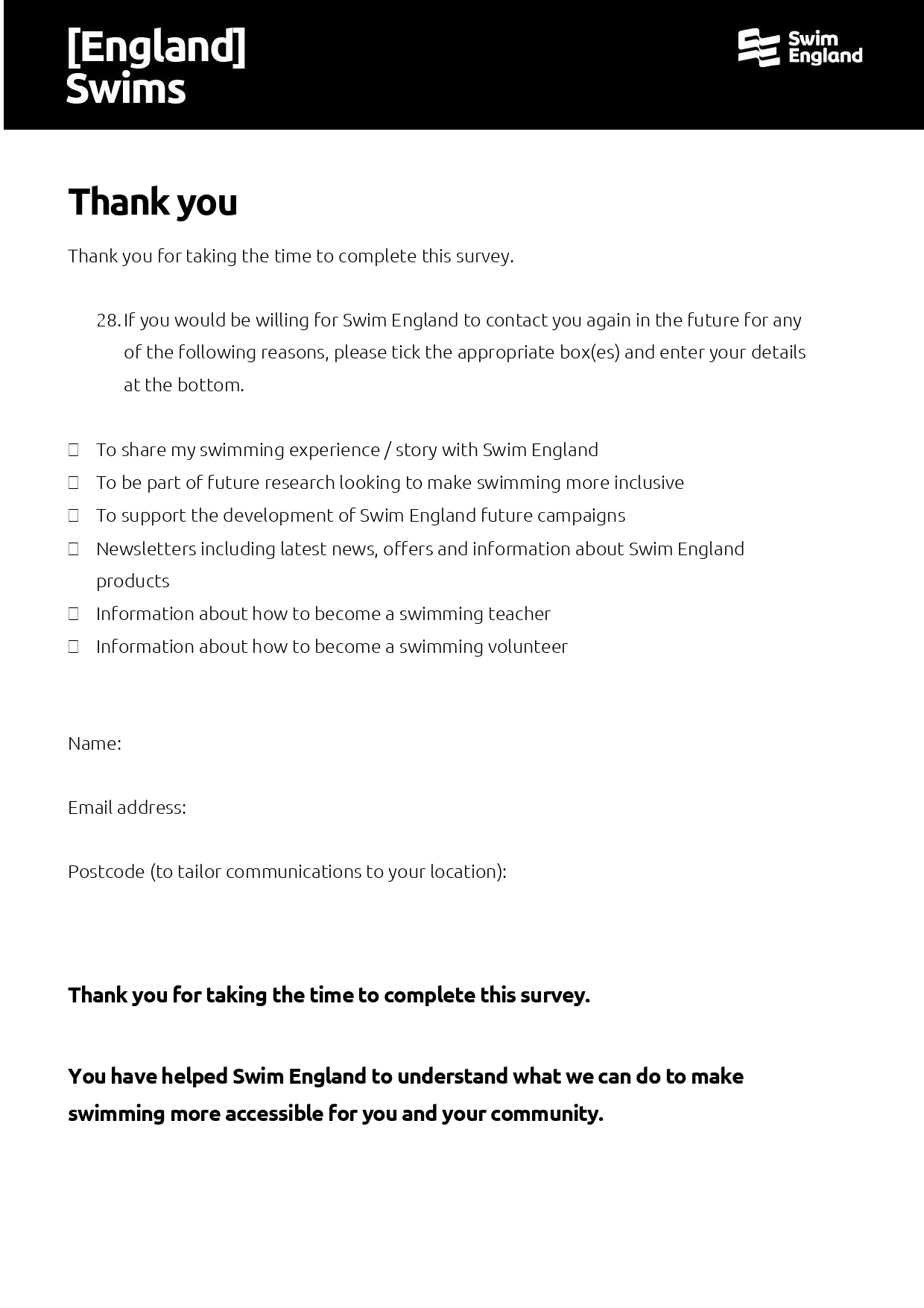

Supplement: ckag075_Supplementary_Data [file ckag075_supplementary_data.zip › ejph-2025-09-om-0744-File006.docx]
