# Supplementary material for: Exploring experiences of swimming and aquatic exercise in individuals with long term health conditions: results from Swim England’s ‘England Swims’ survey
Source: Eur J Public Health. 2026 Jul 10;36(4):ckag075. doi: 10.1093/eurpub/ckag075 (PMC13349663; doi:10.1093/eurpub/ckag075)
Supplement: ckag075_Supplementary_Data [file ckag075_supplementary_data.zip › ejph-2025-09-om-0744-File008.docx]

**Supplementary file S3**

**FREE-TEXT RESPONSES**

| **Theme** | **Comment** | **Age** | **Gender** | **Ethnicity** | **Religion** |
| --- | --- | --- | --- | --- | --- |
| Confidence | *“I can't swim so would look stupid dipping my toes in” (R242)* | 42-57 | Woman | White British, English, Welsh, Scottish, or Northern Irish | Christian (including Church of England, Catholic, Protestant and all other Christian denominations) |
| Confidence | *“Competitive swimmers should have own time/lane. I am not confident in water but can swim. Competitive swimmers are impatient and overtake, making me afraid of drowning” (R362)* | 42-57 | Woman | Any other Black, Black British, or Caribbean background | No religion |
| Confidence | *“I am physically disabled and I would like sessions where I had a lane to myself so I'm not worried about people bumping into me and hurting me” (R619)* | 58-65 | Woman | Irish | Christian (including Church of England, Catholic, Protestant and all other Christian denominations) |
| Confidence | *“My children had a bad experience of drowning but luckily they have been rescued by guides, after that, I am scared of go swimming and so are my kids” (R392)* | 26-35 | Woman | Pakistani | Muslim |
| Confidence | *“Sometimes swimming can get lonely so that shatters my motivation to keep doing it*” *(R718)* | 16 - 25 | Man | African | Christian (including Church of England, Catholic, Protestant and all other Christian denominations) |
| Confidence | *“Need someone to go with me. Am nervous of falling around the pool” (R137)* | 42-57 | Woman | White British, English, Welsh, Scottish, or Northern Irish | Christian (including Church of England, Catholic, Protestant and all other Christian denominations) |
| Confidence | *“I used to go swimming once a week before the Covid pandemic… I now lack the confidence to return as I'm still worried about catching Covid” (R345)* | 66-75 | Woman | White British, English, Welsh, Scottish, or Northern Irish | Christian (including Church of England, Catholic, Protestant and all other Christian denominations) |
| Fear of discrimination, stigma or unwanted attention | “*I'm obese, disabled and use walking aids… I hate how I look, and don't feel comfortable in very little clothing or getting to and from the pool” (R580)* | 36-41 | Woman | White British, English, Welsh, Scottish, or Northern Irish | No religion |
| Fear of discrimination, stigma or unwanted attention | *“I have a visible skin condition and fear that people will judge and I will be made to feel uncomfortable” (R54)* | 42-57 | Woman | Any other Black, Black British, or Caribbean background | No religion |
| Fear of discrimination, stigma or unwanted attention | “*I feel that I'm judged for being slow and I'm in the way, and that if I did need help, no one would know how to help me” (R580)* | 36-41 | Woman | White British, English, Welsh, Scottish, or Northern Irish | No religion |
| Fear of discrimination, stigma or unwanted attention | *“As a fat person I don't feel welcome in swimming pools…. I don't believe that instructors or lifeguards would try to help me if I was having difficulties… I loved swimming when I was a kid, but as an adult I've only had poor experiences” (R586)* | 42-57 | Woman | White British, English, Welsh, Scottish, or Northern Irish | No religion |
| Fear of discrimination, stigma or unwanted attention | *“The local pool isn't inclusive. They have repeatedly ignored or made excuses when asked to provide inclusive swimming sessions with female lifeguards. We have been requesting their assistance for over 6 years to no avail” (R418)* | 42-57 | Woman | Indian | Muslim |
| Fear of discrimination, stigma or unwanted attention | *“Often changing rooms are communal and Muslim men and women are not allowed to take their clothes off in front of others even if of the same sex. I have to miss 10 [minutes] of the session so I can change in the shower cubicle to avoid the queue as all the other Muslim ladies have to do the same as we cannot use the communal changing rooms” (R397)* | 42-57 | Woman | White British, English, Welsh, Scottish, or Northern Irish | Muslim |
| Fear of discrimination, stigma or unwanted attention | *“I'm trans and early in my transition - it's hard to navigate changing rooms without scaring/bothering/upsetting others” (R17)* | 26-35 | Transgender man | Any other White background | Jewish |
| Fear of discrimination, stigma or unwanted attention | *“I have experienced transphobic harassment from other swimmers and pool staff in the past” (R543)* | 36-41 | Prefer to self describe | White British, English, Welsh, Scottish, or Northern Irish | Buddhist |
| Availability | *“There are no public swimming sessions that I can just turn up to... I used to enjoy swimming straight after work, but since the pandemic, the local pools don't open for a public swim at these times” (R201)* | 42-57 | Woman | White British, English, Welsh, Scottish, or Northern Irish | Prefer not to say |
| Availability | *“Pool operators do not value recreational swimmers enough. Evening programs focus on clubs and Learn to Swim. Some people requiring low-impact activities are not old and work during the daytime so evening access is very important” (R648)* | 36-41 | Woman | White British, English, Welsh, Scottish, or Northern Irish | No religion |
| Availability | *“My local pool closed down and hasn't reopened therefore to travel to my nearest pool would prove too expensive and time consuming“ (R124)* | 42-57 | Woman | White British, English, Welsh, Scottish, or Northern Irish | Christian (including Church of England, Catholic, Protestant and all other Christian denominations) |
| Availability | *“I’m on a low income but am not eligible for any benefits. All my money goes on the bills... so swimming is too expensive“ (R420)* | 42-57 | Woman | Pakistani | Muslim |
| Availability | *“Cost of taking a family each time is £10 a week or £40 a month minimum. Family monthly passes need to be introduced“ (R295)* | 42-57 | Prefer not to say | Any other White background | Prefer not to say |
| Availability | *“I would like to swim outdoors more, in a safe space that is close by, but all the lidos have closed” (R457)* | 36-41 | Woman | White British, English, Welsh, Scottish, or Northern Irish | No religion |
| (Un)welcoming environment | *“Women who are survivors of sexual violence and women from religious communities are being driven away from swimming by the absolute failure to recognise our needs to single sex services…” (R651)* | 42-57 | Woman | Any other South Asian background | Sikh |
| (Un)welcoming environment | *“It is important for women to feel safe both physically and socially. A pool and changing room is a place where women and girls can feel particularly vulnerable, due to actual or near nakedness” (R653)* | 42-57 | Woman | White British, English, Welsh, Scottish, or Northern Irish | No religion |
| (Un)welcoming environment | *“I’m a Muslim woman and the ladies only sessions are either not at suitable times or too crowded and they don't cater for women who can actually swim” (R281)* | 36-41 | Woman | White British, English, Welsh, Scottish, or Northern Irish | Muslim |
| (Un)welcoming environment | *“Lack of male only and women only sessions with the corresponding same sex lifeguard in a clean not overlooked pool without members of the public or staff being able to walk in” (R397)* | 42-57 | Woman | White British, English, Welsh, Scottish, or Northern Irish | Muslim |
| (Un)welcoming environment | *“Pool is run down, water too cold, closed too often, changing rooms are revolting… as are the other facilities- mould mildew, holes, broken and smell and toilets are even more disgusting and rarely work, pool overcrowded” (R378)* | 42-57 | Woman | White British, English, Welsh, Scottish, or Northern Irish | Christian (including Church of England, Catholic, Protestant and all other Christian denominations) |
| (Un)welcoming environment | *“Disabled changing rooms are not safe to use” (R260)* | 36-41 | Woman | White British, English, Welsh, Scottish, or Northern Irish | Christian (including Church of England, Catholic, Protestant and all other Christian denominations) |
| (Un)welcoming environment | “*Getting too cold in the water as I have fibromyalgia” (R43)* | 42-57 | Woman | White British, English, Welsh, Scottish, or Northern Irish | No religion |
| (Un)welcoming environment | *“I have CES [cauda equina syndrome] and worry about the temperature of the pool in case by back goes into spasms if the water is too cold” (R85)* | 42-57 | Woman | White British, English, Welsh, Scottish, or Northern Irish | No religion |
| (Un)welcoming environment | *“I have anorexia and I get cold really easily and being in water makes me really cold” (R165)* | 26-35 | Woman | White British, English, Welsh, Scottish, or Northern Irish | No religion |
| (Un)welcoming environment | *“The pools are too chlorinated. If it was at lower levels, I would be able to go for an hour or so but as it stands, I can't even breathe walking past” (R589)* | 26-35 | Non-binary | Irish | No religion |
| (Un)welcoming environment | *“The local swimming pool does not offer quiet times for family swimming for those with autism (e.g. SEN [special educational needs] sessions). Most family times are too busy and too noisy” (R257)* | Prefer not to say | Woman | Any other White background | Prefer not to say |
| (Un)welcoming environment | *“Lack of disability awareness among staff, lack of understanding of autism among staff, lack of physical access, lack of information about accessibility, disabled people frequently get forgotten about and left out of conversations” (R539)* | 36-41 | Woman | White British, English, Welsh, Scottish, or Northern Irish | Christian (including Church of England, Catholic, Protestant and all other Christian denominations) |
| (Un)welcoming environment | *“Local pools won't let me swim due to my mobility/health issues due to not having enough lifeguards or I am not a strong enough swimmer to be in the pool (I can swim at least 2 or 3 lengths without stopping at the moment)” (R121)* | 42-57 | Woman | White British, English, Welsh, Scottish, or Northern Irish | No religion |
